# Mitochondrial dysfunction in an Opa1^Q285STOP^ mouse model of dominant optic atrophy results from Opa1 haploinsufficiency

**DOI:** 10.1038/cddis.2016.160

**Published:** 2016-07-28

**Authors:** Y Kushnareva, Y Seong, A Y Andreyev, T Kuwana, W B Kiosses, M Votruba, D D Newmeyer

**Affiliations:** 1 Immune Regulation, La Jolla Institute for Allergy and Immunology, 9420 Athena Circle, La Jolla, CA 92037, USA; 2 Department of Pharmacology, University of California San Diego, La Jolla, CA 92093, USA; 3 School of Optometry and Vision Sciences, Cardiff University, Cardiff CF24 4LU, UK; 4 Cardiff Eye Unit, University Hospital Wales, Cardiff CF14 4XW, UK

## Abstract

Mutations in the *opa1* (optic atrophy 1) gene lead to autosomal dominant optic atrophy (ADOA), a hereditary eye disease. This gene encodes the Opa1 protein, a mitochondrial dynamin-related GTPase required for mitochondrial fusion and the maintenance of normal crista structure. The majority of *opa1* mutations encode truncated forms of the protein, lacking a complete GTPase domain. It is unclear whether the phenotype results from haploinsufficiency or rather a deleterious effect of truncated Opa1 protein. We studied a heterozygous Opa1 mutant mouse carrying a defective allele with a stop codon in the beginning of the GTPase domain at residue 285, a mutation that mimics human pathological mutations. Using an antibody raised against an N-terminal portion of Opa1, we found that the level of wild-type protein was decreased in the mutant mice, as predicted. However, no truncated Opa1 protein was expressed. In embryonic fibroblasts isolated from the mutant mice, this partial loss of Opa1 caused mitochondrial respiratory deficiency and a selective loss of respiratory Complex IV subunits. Furthermore, partial Opa1 deficiency resulted in a substantial resistance to endoplasmic reticulum stress-induced death. On the other hand, the enforced expression of truncated Opa1 protein in cells containing normal levels of wild-type protein did not cause mitochondrial defects. Moreover, cells expressing the truncated Opa1 protein showed reduced Bax activation in response to apoptotic stimuli. Taken together, our results exclude deleterious dominant-negative or gain-of-function mechanisms for this type of Opa1 mutation and affirm haploinsufficiency as the mechanism underlying mitochondrial dysfunction in ADOA.

The optic atrophy 1 (Opa1) protein is a mitochondrial dynamin-related GTPase required for mitochondrial fusion and the formation of mitochondrial cristae. These core Opa1 functions are essential for mitochondrial bioenergetic competence, mitochondrial DNA stability, control of apoptosis, and possibly autophagy.^1, 2, 3, 4, 5, 6, 7, 8, 9, 10^ Additionally, recent studies demonstrate that Opa1 is important for the regulation of Ca^2+^ homeostasis.^4, 11, 12^ Certain *opa1* mutations lead to autosomal dominant optic atrophy (ADOA), a hereditary eye disease caused by the selective loss of retinal ganglion cells (RGCs) and degeneration of the optic nerve. More than 200 heterozygous *opa1* mutations are cataloged in the current literature. These mutations are distributed along the entire protein coding sequence, but many are clustered in the GTPase domain (reviewed in refs. 13, 14, 15).

Most human *opa1* mutations (typically, nonsense and frameshift) are predicted to encode truncated transcripts.^14, 15, 16^ Generally, transcripts with premature termination codons are prone to degradation via nonsense-mediated mRNA decay^17^ and could therefore produce haploinsufficiency. Indeed, it has been reported that truncated transcripts often constitute less than the expected 50% of the total pool of *opa1* transcripts. However, the extent of depletion of various mutant *opa1* transcripts in ADOA patients is highly variable, ranging from no change to an apparent ~2/3 loss.^18^ It is unclear whether truncated Opa1 proteins expressed from these shortened transcripts are present at all within cells, and if so, whether they have a dominant effect. A more severe ‘ADOA plus syndrome'^14, 15, 19^ arises from a group of pathological point mutations in the GTPase domain. The affected patients exhibit multiple neuromuscular defects, in addition to the loss of RGCs and other classical ADOA symptoms. It has been hypothesized that dominant-negative or deleterious gain-of-function effects (as opposed to haploinsufficiency) account for the pathogenesis in these cases.^14^

Animal models for ADOA have been useful experimental systems for broader functional studies of Opa1 mutations. Davies *et al.*^20^ generated an Opa1 mutant mouse carrying a defective allele with a stop codon at residue 285 (Q285STOP) at the beginning of the GTPase domain. This mutation mimics human pathological mutations in this region.^21^ The heterozygous Opa1^Q285STOP^ mice have normal longevity, but gradually develop visual dysfunction, resembling a slow onset of the human disease. This mild ADOA-like phenotype is typically manifested at 6–9 months and becomes more prominent in aged animals. The delayed phenotype suggests that the Opa1 mutations lead to a chronic accumulation of mitochondrial damage, possibly in synergy with effects of aging. In contrast to this slow phenotypic onset in heterozygous animals, the homozygous mutant embryos are already malformed at E9.5 and die before E14.5.^20^ Early embryonic lethality is also observed in homozygotes from other mouse ADOA models.^22, 23^

In the heterozygous Opa1^Q285STOP^ mice, mutant and normal transcripts are present at the same level. As expected, full-length (wild-type (WT)) Opa1 protein is reduced by ~50% in most tissues tested, but it is not known whether truncated mutant polypeptides are produced.^20, 21^ In Opa1^Q285STOP^ cardiomyocytes, age-dependent reductions in the activities of electron transport chain (ETC) Complexes I and IV were observed, possibly linked to mitochondrial DNA (mtDNA) instability.^24^ However, in the skeletal muscle, no changes in mtDNA levels and Complex IV activity were detected,^25^ implying that the mitochondrial defects are tissue-specific.

Major loss of Opa1 function (e.g., in cells treated with *opa1* siRNA) typically sensitizes cells to mitochondrial apoptosis,^2, 3, 4^ whereas Opa1 overexpression has a moderate protective effect.^8, 9, 26^ Opa1 complexes have a role in keeping mitochondrial crista junctions in a closed state, limiting cytochrome *c* mobilization from the crista interior. Thus, it is important for apoptotic cells to disassemble Opa1 complexes, to allow complete release of soluble proteins from the cristae during apoptosis.^8, 9^ Indeed, we showed that the overexpression of a mutant Opa1 with enhanced self-assembly strongly reduces Bax/Bak-induced cytochrome *c* release into the cytosol.^8^ This inhibition of Opa1 disassembly does not affect Bax translocation and activation in the mitochondrial outer membrane (MOM), which occur upstream and independently of crista remodeling events.^8, 9^ A recent study showed that the disassembly of Opa1 complexes and cytochrome *c* release require proteolytic cleavage of Opa1 by the mitochondrial metalloprotease Oma1.^27^

It is also conceivable that reduced Opa1 function could sensitize cells to apoptosis and other forms of cell death indirectly, by compromising ATP levels and overall mitochondrial bioenergetic integrity. With regard to ADOA, it remains unclear whether Opa1 mutations promote the disease through effects on apoptosis. Some studies found no evidence for increased apoptotic death in human cells harboring ADOA-linked mutations.^28, 29^ In RGCs, decreased Opa1 function was also associated with autophagic^7, 30^ and excitotoxic^4, 31^ cell death pathways. In both cases, cell death is caused at least partly by mitochondrial bioenergetic defects.

Here we investigated the pathological mechanism underlying mitochondrial dysfunction in the Opa1^Q285STOP^ ADOA mice. When we examined mouse embryonic fibroblasts (MEFs) from these animals, we found a bioenergetic deficiency, as well as the decreased expression of components of cytochrome *c* oxidase (COX) consistent with reduced enzymatic COX activity. The respiratory deficiency increased as the cells were passaged. Further, we found that the predicted Opa1 truncation mutant protein was undetectable in MEFs and tissues from the animals. This implies that haploinsufficiency, rather than a dominant-negative effect of truncated Opa1 protein, is the primary mechanism for the ADOA-like phenotype. We therefore suggest that the human disease may also result from Opa1 haploinsufficiency. Interestingly, we found that Opa1^Q285STOP^ MEFs are relatively resistant to apoptosis induced by endoplasmic reticulum (ER) stressors, while showing increased or normal sensitivity to other apoptotic stimuli. This suggests a novel functional link between Opa1 and ER stress-induced mitochondria-dependent cell death. To compare the phenotypic effects of Opa1 haploinsufficiency *versus* concurrent expression of truncated and WT protein, we enforced the expression of truncation mutants in cultured cells. We found that mitochondrial respiration was not impaired, again suggesting that mitochondrial functional defects arise from the lack of sufficient Opa1 function rather than a dominant-negative effect of truncated protein. Surprisingly, the expression of mitochondria-targeted truncated Opa1 mutants inhibited apoptotic Bax activation. This suggests that the N-terminal portion of WT Opa1 could be involved in apoptotic signaling from the inner mitochondrial membrane outward to the MOM or cytoplasm.

## Results and Discussion

### Bioenergetic function is impaired in late-passage Opa1 mutant mouse embryo fibroblasts

**Figure 1:**
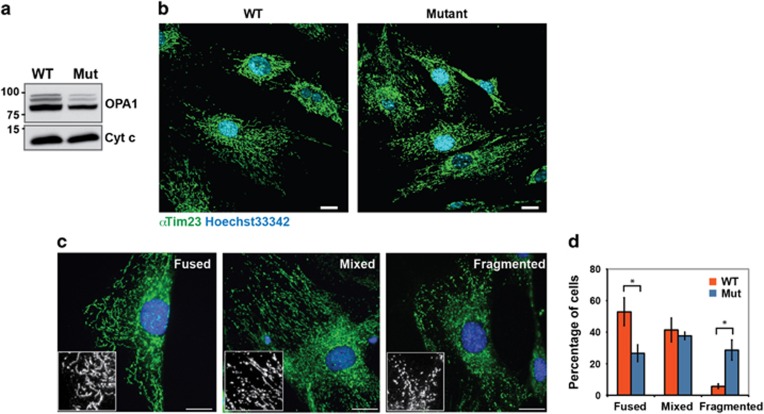
Opa1 protein level and mitochondrial morphology in MEFs isolated from the Opa1^Q285STOP^ mouse. (**a**) Opa1 protein level is reduced in Opa1^Q285STOP^ MEFs. Cytochrome *c* (Cyt *c*) is shown as a loading control. (**b**) Mitochondrial morphology in WT and Opa1^Q285STOP^ MEFs. Scale bar, 20 *μ*m. (**c**) Representative images of different mitochondrial phenotypes: elongated, mixed (heterogeneous), and fragmented mitochondria. Scale bar, 20 *μ*m. (**d**) Quantification of the different phenotypes in WT and mutant MEFs. Data are means±S.E.M. from six independent cell cultures/plates combined from two independent preparations of MEFs

Opa1^Q285STOP^ mice have been characterized in terms of visual dysfunction, neuromuscular defects, optic nerve atrophy, and RGC abnormalities.^20, 21^ However, published data on mitochondrial function in this ADOA model are inconclusive. In one study, estimation of the membrane potential in isolated brain and retinal mitochondria (using JC-1 dye) showed no changes in the mutant samples, even though the authors observed abnormal mitochondrial ultrastructure with reduced numbers of cristae.^32^ In contrast, a significant decrease in respiratory function was reported for isolated heart mitochondria.^24^ To analyze consequences of the Q285STOP mutation, we isolated MEFs from heterozygous animals. We first confirmed the predicted ~50% loss of full-length (WT) Opa1 protein by immunoblot (Figures 1a and 3). The levels of long and short Opa1 isoforms were reduced to a similar extent, indicating that Opa1 processing was not affected (Figure 1a).

**Figure 2:**
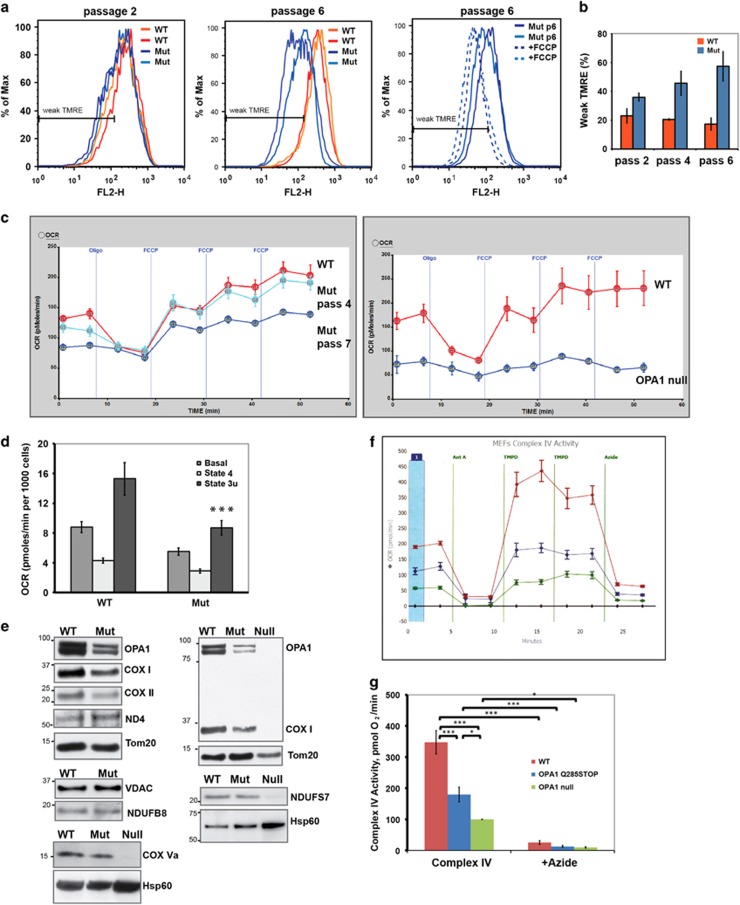
Delayed impairment in bioenergetic function and decreased expression of Complex IV in Opa1^Q285STOP^ MEFs. (**a** and **b**) Assessment of the mitochondrial membrane potential by TMRE staining and flow cytometry: (**a**) A significant decline in mitochondrial membrane potential is observed in the Opa1 mutant MEFs (blue lines) at passage 6 but not at passage 2. Right panel shows the effect of FCCP (1 *μ*M) that was used as a control for weak TMRE staining (low membrane potential). (**b**) Quantification of the TMRE staining data. Data shown are percentage of cells with weak TMRE staining. Error bars are S.D. from three to four independent measurements. (**c** and **d**) Cellular respiration (OCR) was measured in intact MEFs using Seahorse Biosciences Inc. technology. (**c**) Representative results obtained for WT and Opa1 mutant MEFs at different passages. Output panels of the Seahorse XF24 Flux Analyzer are shown, each representing ‘raw' data from an individual 24-well plate. Vertical lines indicate timing of injection of oligomycin (2 *μ*g/ml) and sequential injections of FCCP (600 nM) to induce resting (State 4) and maximal (state 3u) respiration, respectively. Data are means±S.E. from five to eight replicate wells. OCRs were significantly reduced in Opa1 mutant MEFs at passage 7 (left panel). Opa1-null MEFs exhibited a nearly complete loss of respiration and were used as a positive control (right panel). (**d**) Summary of respiration measurements (seven experiments similar to one shown in (**c**) were performed and averaged). Basal (before additions), State 4 (resting, oligomycin-inhibited) and state 3 u (maximal FCCP-induced) OCR in WT and late-passage (between 7 and 10) mutant MEFs are shown. Data are means±S.E. from seven independent cell cultures/plates. (**e**) Western blot analysis of ETC subunits in whole- cell lysates prepared from WT and Opa1 mutant MEFs. Some blots also include samples from Opa1-null MEFs for comparison. The levels of Complex IV subunits (COX I and COX II) were reduced in Opa1 mutant MEFs while there were no decreases in the levels of Complex I subunits (ND4, NDUFB8, and NDUFS7). The levels of COX I subunit are shown on two immunoblots (from two independent cell lysate preparations). VDAC, Hsp60 or Tom20 remained unchanged in Opa1 mutant MEFs. The membranes from each individual blot were probed with an Opa1 antibody to verify the partial loss of Opa1 in mutant samples (see also Supplementary Figure S1). (**f** and **g**) Complex IV activity measured in MEFs permeabilized with perfringolysin O (PFO). (**f**) Results of a representative experiment shown as the Seahorse XF^e^96 Flux Analyzer output panel. Vertical lines indicate following injections: 1 *μ*M antimycin A (Ant A); 2.5 mM TMPD and 5 mM ascorbate (TMPD); 20 mM NaN_3_ (azide). (**g**) Summary results of three experiments. Complex IV activity was measured as OCR in the presence of TMPD (as shown on panel f). Note that this reaction was almost completely inhibited by addition of NaN_3_ (‘+ azide'). Data are mean±S.E.M. ***, **, and * statistically significant differences with *P*<0.001, *P*<0.01, and *P*<0.05, respectively

In individual WT and mutant cells, mitochondrial network morphologies fell into three categories: heterogeneous, mostly elongated (fused), or mostly fragmented (Figures 1b and c). Quantification of the different phenotypes showed that on average, mitochondria were more fragmented in the heterozygous Opa1^Q285STOP^ mutant cells than in WT cells (Figure 1d). This is consistent with a partial loss of Opa1-dependent mitochondrial fusion. However, a significant fraction of mutant cells contained elongated mitochondria, which suggests that even halved levels of WT Opa1 can support mitochondrial fusion substantially. Total mitochondrial mass in these cells was likely preserved, as we detected normal levels of the matrix marker Hsp60 and membrane markers Tom20 and VDAC (Figure 2e).

Superficially, the loss of 50% of Opa1 protein appeared not to affect cell function, as WT and Opa1 mutant MEFs displayed similar growth patterns that are typical for primary MEF cultures:^33^ after initial rapid proliferation, cell division started to decline after 4–5 passages and virtually stopped after 8–10 passages. Moreover, in early passage cells, no bioenergetic changes were detected. However, in mutant cells at later passages, we observed decreased mitochondrial membrane potential (Figures 2a and b) and oxygen consumption rates (OCRs), compared with WT (Figures 2c and d). Oligomycin-inhibited OCR (state 4), an indicator of proton leak through the inner membrane, was not elevated in mutant cells (Figures 2c and d). Also, the rates of ADP-induced (state 3) respiration measured *in situ* in cells with selectively permeabilized plasma membrane (to allow the access of exogenously added ADP to mitochondria) were reduced in Opa1 mutant cells (Supplementary Figure S1A). This demonstrates that the decrease in the membrane potential was caused by partial respiratory inhibition and not by mitochondrial uncoupling.

The bioenergetic defect can be explained by a functional deficiency of Complex IV (COX), as the levels of the mitochondrial-encoded subunits COX I and COX II, but not nuclear-encoded COX Va subunit, were lower in Opa1 mutant MEFs than in control cells (Figure 2e and Supplementary Figure S1B). Furthermore, Opa1 mutant MEFs showed significantly reduced enzymatic activity of Complex IV, assayed as oxidation of its artificial specific electron donor TMPD (*N*,*N*,*N′*,*N*'-tetramethyl-*p*-phenylene diamine) by measuring OCR (Figures 2f and g). Our observations of reduced COX protein levels and activity in Opa1^Q285STOP^ MEFs are consistent with published functional and histological evidence for COX deficiency in cells with various Opa1 mutations.^28, 34^

Because levels of mitochondrial- but not nuclear-encoded Complex IV protein levels were reduced in Opa1 mutant MEFs, our results raise the possibility that Opa1 haploinsufficiency leads to a partial loss of mtDNA. Further study will be needed to test this. However, arguing against this potential mechanism are reports in the literature: for example, a study of skin fibroblasts from ADOA patients reported decreased COX enzymatic activity in the absence of detectable changes in the amount of mtDNA.^28^ Similarly, a mtDNA-independent decrease in COX (but not Complex I) activity was observed in mice lacking Opa1 in pancreatic *β*-cells.^35^ In cells with a more profound loss of Opa1, we saw an even more severe respiratory deficiency. Immortalized *opa1*-null MEFs^6^ showed a nearly complete loss of mitochondrial respiration, major depletion of both mitochondrial- and nuclear-encoded ETC components, and a significant decrease in the level of Tom20 (Figure 2e and Supplementary Figure S1B).

Recent studies highlighted a role of Opa1-dependent crista integrity in the assembly of individual ETC complexes into higher order supercomplexes (e.g., Complex I–III, Complex I–III–IV, and Complex III–IV) that may enhance ATP production.^36^ In particular, acute short-term Opa1 ablation preserved mtDNA levels and translation of mitochondrially encoded subunits, but, nevertheless, led to compromised mitochondrial bioenergetic efficiency, which correlated with deficient assembly of ETC supercomplexes.^5^ It was not entirely clear to what degree this defect affected total levels of individual active respiratory complexes. Further study will be needed to determine whether Opa1 haploinsufficiency affects the assembly of respiratory supercomplexes in cells from Opa1^Q285STOP^ mice.

Ordinarily, COX is expressed at levels conferring significant functional excess compared with other ETC complexes, which may explain the typically subtle or absent respiratory defects in cells with heterozygous Opa1 mutations. However, an earlier bioenergetic study argued that the functional excess of COX activity in whole cells (as opposed to isolated mitochondria) does not exceed 20–40%.^37^ The threshold level of COX required for normal respiration may be variable between cell types and affected by oxygen and substrate availability, cytochrome *c* pools, and ATP demand.^37, 38^ Thus, the ~40% COX reduction seen in late-passage Opa1^Q285STOP^ MEFs might cause COX to become rate limiting for ETC function. One can also hypothesize that in RGCs and other relevant cells, a lower spare respiratory capacity of COX or other ETC components (rather than protein level *per se*) makes them more vulnerable to pathogenic Opa1 mutations.

### The truncated form of Opa1 is not expressed in the Opa1^Q285STOP^ mouse

It is still not fully understood whether the genetic dominance of ADOA results from haploinsufficiency or a dominant effect of the mutant protein. As noted above, in the Opa1^Q285STOP^ mouse, mutated Opa1 transcript is not degraded.^20^ We considered the possibility that the N-terminal Opa1 fragment is expressed and active in some way. To test this, we analyzed MEFs and tissues from the Opa1 mutant mice by immunoblot, using an affinity-purified antiserum raised against a recombinant Opa1_114–289_ polypeptide, which recognizes the truncated forms of Opa1. Also, we generated HeLa cell lines with doxycycline (Dox)-inducible expression of a nearly identical FLAG-tagged Opa1 truncation mutant (Opa1_1–289_; Figure 5A).

**Figure 3:**
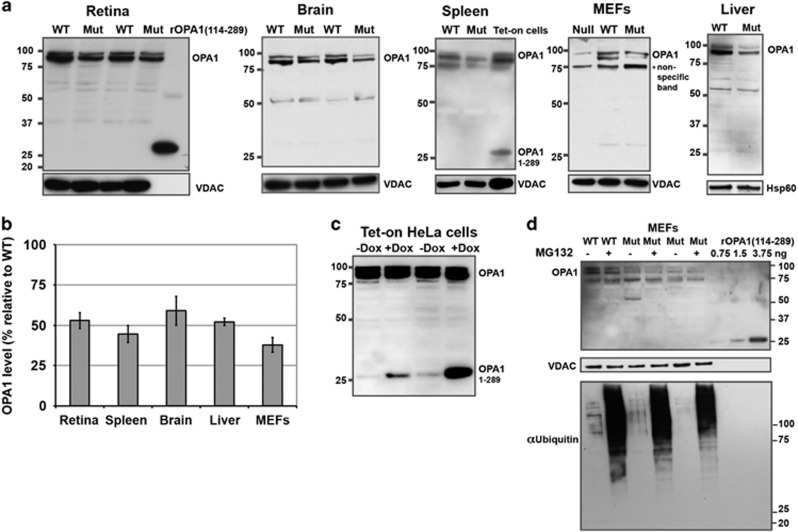
Mutated Opa1 is not expressed at detectable levels in Opa1^Q285STOP^ mice. (**a**) Samples of indicated tissues derived from the Opa1^Q285STOP^ mice were probed for the presence of Opa1_1–285_ protein by western blot analysis using Opa1 antisera generated against an N-terminal Opa1 polypeptide. As a positive control for the specific antisera reactivity, samples of HeLa cells with doxycycline-inducible expression of Opa1_1–289_ (also shown in panel c) or the recombinant Opa1 polypeptide (rOpa1_114–289_) were loaded on the gels, where indicated. As a negative control for antibody crossreactivity, a sample of Opa1-null cells was added, where indicated. Samples were loaded at 30–50 *μ*g per lane. Endogenous truncated Opa1 was not detected in any of the samples tested (including isolated liver mitochondria), whereas WT Opa1 and inducibly expressed Opa1_1–289_ were readily detected by the Opa1 antisera. (**b**) Quantification of Opa1 protein level confirms its ~50% reduction in all mutant samples. (**c**) Doxycycline-induced Opa1_1–289_ expression in HeLa cells one day (second lane) or 2 days (last lane) after the addition of doxycycline. Whole-cell lysates were loaded at 30 *μ*g per lane. (**d**) Proteasomal inhibition by MG132 does not lead to accumulation of endogenous Opa1_1–285_. MEFs were pretreated with 2 *μ*M MG132 for 24 h before cell lysate preparation. (Last three lanes on the blot contained the recombinant Opa1 polypeptide loaded at indicated amounts.) The membrane was reprobed with ubiquitin antibody to confirm accumulation of ubiquinated proteins and VDAC antibody for a loading control

Whereas inducibly expressed Opa1_1–289_ protein in cultured cells was readily detected by our antibody, endogenous Opa1_1–285_ protein was not detected in any of the samples (including liver mitochondria) derived from the mutant mice (Figures 3a and c). As expected, the level of WT Opa1 in mutant mice was reduced by nearly half in all tissues tested (Figure 3b). Because the predicted truncated Opa1 mutant protein was undetectable, we conclude that the bioenergetic defects (Figure 2) and other previously described abnormalities in the Opa1^Q285STOP^ mouse^21^ arise purely from haploinsufficiency of WT Opa1. A prior study of another mouse ADOA model with a different mutation (Opa1^329–355del^) similarly detected no shortened Opa1 mutant proteins in various tissues tested.^22^ The mechanisms underlying this absence of truncated Opa1 products in ADOA mutant mice are unknown. Degradation of truncated Opa1 by canonical proteasomes is probably not involved, as we found that pretreatment of mutant MEFs with MG132, a proteasome inhibitor, did not cause the mutant protein to appear (Figure 3d).

### Effect of the Opa1 mutation on apoptosis depends on the type of apoptogen

**Figure 4:**
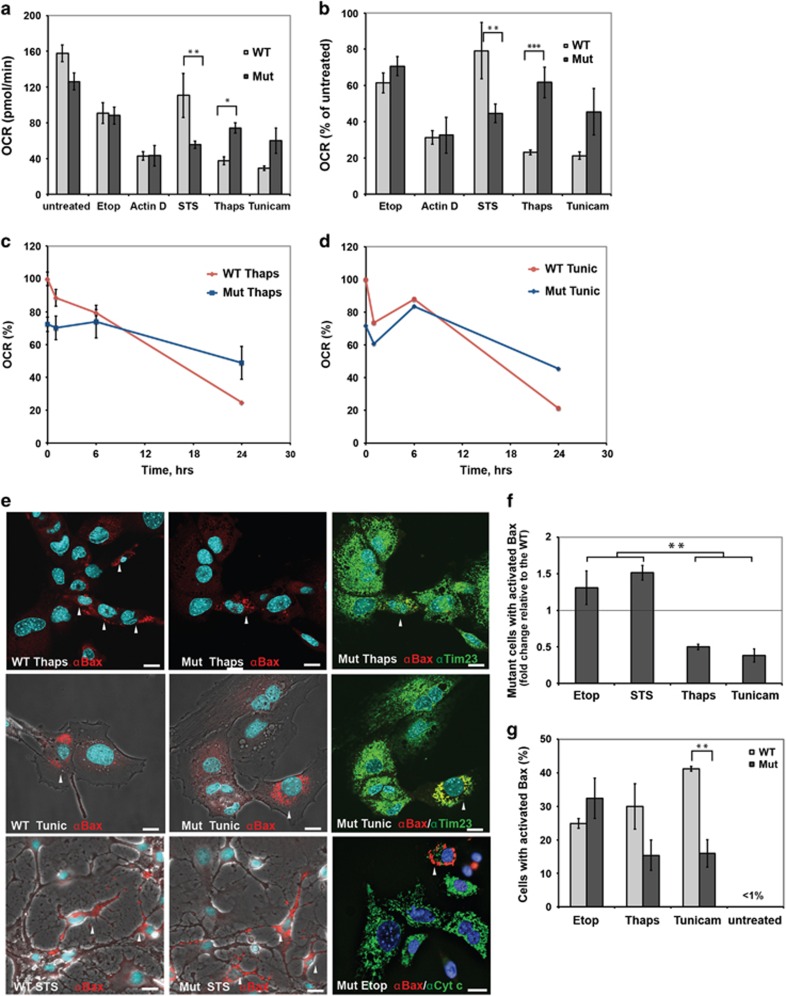
Differential effects of apoptogens on WT and Opa1^Q285STOP^ MEFs. (**a-d**) Maximal respiratory capacity of intact cells (state 3u) treated with various apoptogens was determined in experiments similar to one shown in Figure 2c. (**a**) 300 *μ*M etoposide (Etop), 1 *μ*M actinomycin D (Actin D), 1 *μ*M thapsigargin (Thaps) and 2 *μ*g/ml tunicamycin (Tunic) were added 24 h before the measurements, and STS (1 *μ*M) was added for 5 h. All incubations were performed in the presence of 20 *μ*M Q-VD. (**b**) Relative change in maximal respiratory capacity was calculated from data plotted in (**a**). OCRs of apoptogen-treated WT and mutant MEFs were normalized to OCRs of respective untreated (either WT or mutant) cells in each experiment and averaged. Data shown are means±S.E., *n*=3–7. (**c** and **d**) Time course of the effect of ER stressors on respiration. WT and Opa1 mutant MEFs were treated with thapsigargin (**c**) or tunicamycin (**d**) for 1, 6, and 24 h, and respective OCRs (averaged from five replicate wells for each time point) were normalized to untreated cells at time 0 h. (**e**) Representative images of WT and Opa1 mutant MEFs treated with indicated apoptogens (as in **a**) and immunostained for active Bax (red). Cells were also immunostained for Tim23 or cytochrome *c* (green) as indicated. Nuclei were stained with Hoechst 33342 (blue). Some confocal images are shown with phase contrast to demonstrate cell morphology. Arrows indicate Bax-positive (apoptotic) cells. Scale bar, 20 *μ*m. Bax-positive cells were virtually absent from untreated samples. (**f**) A fold change in the number of apoptotic Opa1 mutant cells relative to the WT. Statistical analysis was performed using one-way ANOVA with *post hoc* Newman–Keuls multiple comparison test. Significant differences between effects of apoptogens are indicated (**P<0.01). Effects of apoptogens (STS, thapsigargin, and tunicamycin) compared with control are also statistically significant (*P*<0.05). (**g**) Percentage of cells with activated Bax among WT and Opa1 mutant MEFs treated with thapsigargin, tunicamycin, or etoposide (as in **a**). Data are means±S.E. from three independent experiments. (For STS, the numbers were variable between experiments and only normalized data are shown in panel f)

Experimental silencing of Opa1 typically increases cellular sensitivity to various apoptotic stimuli.^2, 4, 39, 40^ Increased susceptibility of *opa1*-heterozygous fibroblasts from ADOA patients to cell death has been also reported.^39, 41^ However, changes in apoptotic response are often absent or less pronounced in ADOA cells.^28, 29^ Previous studies of retinal and cardiac tissues from the Opa1^Q285STOP^ mutant mouse found no evidence for increased basal frequencies of apoptosis,^21, 24^ but the effects of agents that stimulate apoptosis (apoptogens) have not been tested in this ADOA model. We compared the responses of WT and Opa1^Q285STOP^ MEFs to various apoptotic stimuli. To prevent rapid deterioration and loss of cells as a result of caspase activation, all apoptogens were added in the presence of a caspase inhibitor (Q-VD). As mitochondrial function is ultimately compromised in cells that have undergone MOM permeabilization (MOMP), even when caspase activity is blocked,^42^ we used mitochondrial respiration as a measure of the viability and bioenergetic competence of apoptogen-treated cells. As expected, all of the drugs caused a significant decline in maximal respiratory capacity (state 3 u or uncoupler-stimulated OCR) in both WT and mutant cells (Figures 4a and b). There were no significant differences between WT and Opa1^Q285STOP^ MEFs in their responses to etoposide or actinomycin D. Thus, a 50% reduction in the Opa1 level did not sensitize cells to death in response to some apoptogens. However, the mutant cells were slightly more sensitive to staurosporine (STS).

Unexpectedly, we found that Opa1 mutant MEFs were more resistant than WT to two inducers of ER stress, tunicamycin and thapsigargin (Figures 4a–d). A 24-h treatment with these agents led to a decline in respiration in WT MEFs, but this effect was blunted in the heterozygous Opa1^Q285STOP^ MEFs (Figures 4a and b). Cell death in response to ER stress typically involves Bax/Bak-dependent MOMP.^43, 44^ To confirm that the observed decline in respiration reflected the occurrence of Bax-dependent apoptosis, we used immunofluorescence microscopy to quantify the percentage of cells stained with antibodies (N20 or 6A7) recognizing activated Bax. Mitochondrial localization of activated Bax was confirmed by costaining with antibodies to Tim23. Apoptotic cells displayed the typical clusters of activated Bax at mitochondria (Figure 4e). Essentially all cells with activated Bax underwent MOMP, as demonstrated by a loss of cytochrome *c* immunostaining (shown for etoposide-treated cells in Figure 4e). In the absence of apoptogens, few cells showed mitochondrial Bax(N20) staining. Overall, results from immunostaining experiments were consistent with the data in Figure 4b. Thapsigargin and tunicamycin treatment produced less apoptosis in Opa1 mutant MEFs than in WT MEFs, whereas the effects of etoposide were similar in WT and Opa1 mutant MEFs (Figures 4f and g). Furthermore, image analysis confirmed increased sensitivity of Opa1 mutant MEFs to STS treatment (Figure 4f).

ER stress typically leads both to a rapid Ca^2+^ pulse released from the ER and to a slower transcriptional response (reviewed in Wang and Kaufman^45^). Although tunicamycin (an inhibitor of N-glycosylation) and thapsigargin (an inhibitor of ER/sarcoplasmic Ca^2+^ pumps) target different ER functions, both of them induce similar sustained mitochondrial Ca^2+^ increases within several minutes.^46^ It is conceivable that early energy-dependent mitochondrial Ca^2+^ uptake is compromised in Opa1 mutant MEFs, as has been demonstrated in other Opa1-deficient cells.^4^ Other recent studies have shown that the MOM fusion proteins Mitofusin 1 and Mitofusin 2 (Mfn1 and Mfn2) can also modulate the cellular response to ER stress. One proposed mechanism involves interaction of Mfn2 with PERK, the ER-resident protein kinase initiating signaling events comprising specific ER stress response.^47^ Another mechanism involves a more general effect in which very small mitochondria are relatively resistant to Bax-mediated MOMP.^44^ In both cases, loss of Mfn1 or Mfn2 function reduced ER stress-mediated apoptosis. Further study will be required to elucidate the mechanisms through which Opa1 haploinsufficiency leads to resistance to ER stress-induced apoptosis.

### Overexpression of Opa1_1–289_ had no effect on mitochondrial morphology and respiration but decreased Bax activation

**Figure 5:**
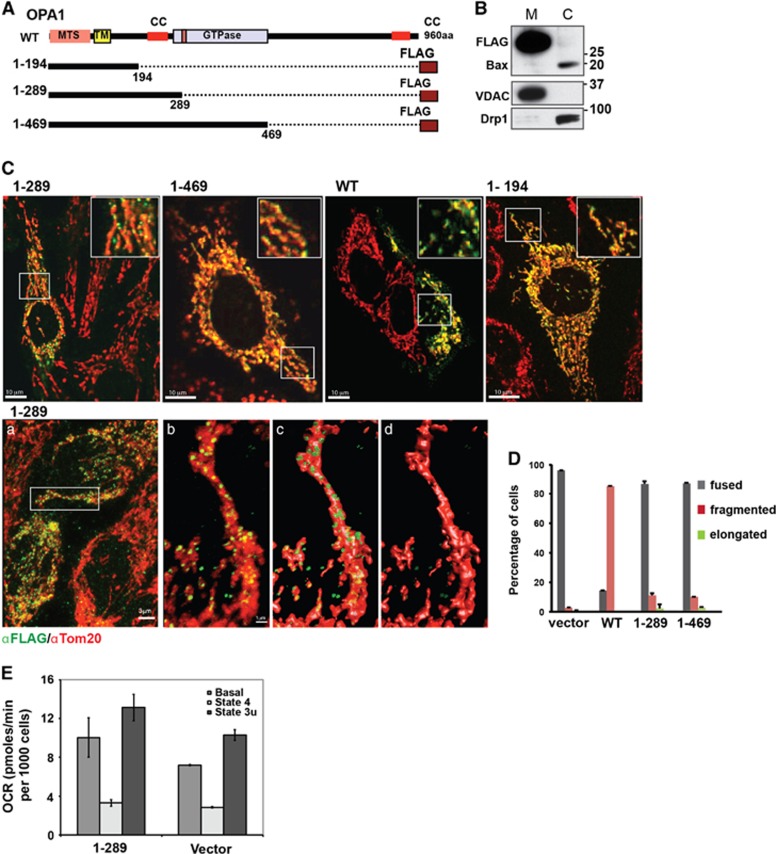
Expression of N-terminal Opa1 mutants has no effect on mitochondrial morphology and function. (**A**) Schematic representation of human Opa1 and the truncated mutants. Full-length wild-type Opa1 contains mitochondrial targeting sequence (MTS), a putative transmembrane domain (TM), two coiled-coil domains (CC), and a dynamin-like GTPase domain (GTPase). The truncated mutant (1–289) has only MTS, TM, and CC. (**B** and **C**) FLAG-tagged Opa1_1–289_ mutant is localized to the mitochondria. (**B**) Western blot of subcellular fractions shows localization of Opa1_1–289_ (detected by FLAG antibody) in heavy membrane (M) fraction; VDAC was used as a marker for the membrane (mitochondria-enriched) fraction, and Bax and Drp1 were the markers for the cytosolic (c) fraction. Samples were loaded at 30 *μ*g per lane. (**C**) Confocal images showing mitochondrial localization of the FLAG-tagged Opa1 mutants (1–289, 1–469, 1–194) and FLAG-tagged WT Opa1. Cells were immunostained with antibodies to FLAG (green) and Tom20 (red) to visualize mitochondrial morphology. Bottom panels also show a higher resolution image of mitochondria containing Opa1_1–289_ (green). A zoomed-in area indicated by white rectangular in panel (a) is shown in panel (b) and its three-dimensional (3D) reconstruction (isosurface rendering) is shown in panels (c and d) with translucent (c) and solidified (d) representation of the mitochondrial surface (red). Areas of colocalization are shown in yellow. See also Supplementary Figure 2. (**D**) Quantification of mitochondrial phenotypes (tubular, fragmented, or elongated) in cells overexpressing WT Opa1 or indicated Opa1 mutants. (**E**) Cellular respiration (OCR) was measured in intact control cells (vector) and cells expressing Opa1_1–289_: basal (before additions), State 4 (resting, oligomycin-inhibited) and state 3u (maximal FCCP-induced) OCR. Data are means±S.D. from three independent cell cultures

As shown above, the mutant Opa1_1–285_ polypeptide was not detected *in vivo* (Figure 3). The repression mechanism for the truncated protein is unknown. Nevertheless, we asked whether a similar truncated Opa1 protein, if expressed, could cause defects in mitochondrial morphology or respiration. We used HeLa cells to generate a cell line with stable Dox-inducible expression of FLAG-tagged Opa1_1–289_ (Figure 5A). (The breakpoint at residue 289 had been chosen for an earlier study, but is very close to the predicted protein truncated at residue 285 in the mutant mice; both would lack the dynamin-like GTP binding domain but retain the coiled-coil domain.) For comparison, we tested two other mitochondria-targeted mutants (Opa1_1–194_ and Opa1_1–469_; Figure 5A) in some experiments.

**Figure 6:**
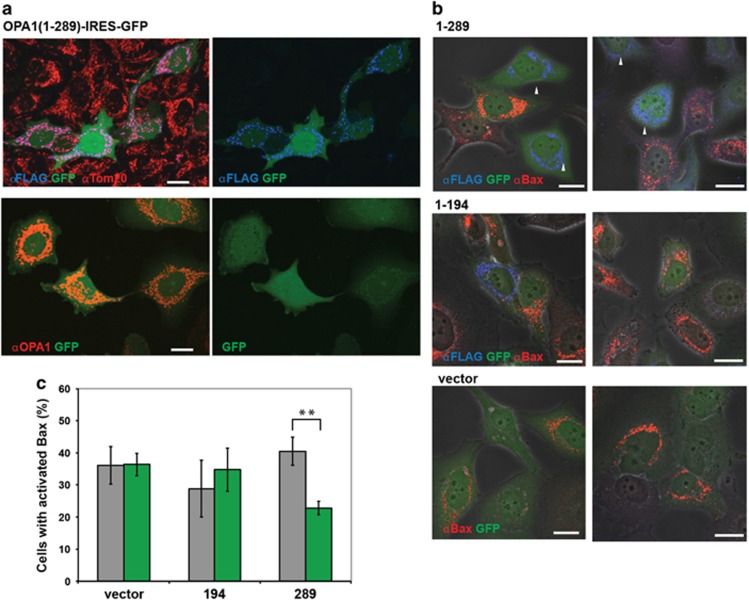
Opa1_1–289_ mutant decreases Bax activation in etoposide-treated cells. (**a**) Dox-induced expression of FLAG-tagged Opa1_1–289_ coupled with the expression of GFP (Opa1_1–289_-IRES-GFP cells). Cells were fixed and immunostained with FLAG and Tom20 antibodies (upper panels) or the N-terminal Opa1 antibody (bottom panels). A representative image shows GFP-positive Opa1_1–289_-expressing cells (green), which display FLAG staining (blue) overlapping with Tom20 staining (red) yielding pink color. Neighboring non-expressing cells (GFP-negative) display only Tom20 staining (red). Bottom panels show representative cells with modest (~2-fold) expression levels of Opa1_1–289_. Scale bar, 20 *μ*m. (**b**) Representative images of etoposide-treated Opa1_1–289_, Opa1_1–194_, and vector control cells (two fields are shown for each cell type as indicated). Expression of the indicated Opa1 mutants was coupled with the expression of GFP (as shown in **a**). Cells were fixed and immunostained with FLAG (blue) and Bax(N20) (red) antibodies after 24 h treatment with 300 *μ*M etoposide in the presence of 20 *μ*M Q-VD. Arrows show examples of GFP-positive cells that have not undergone apoptosis and do not show Bax activation. Scale bar, 20 *μ*m. (**c**) Quantification of Bax activation among GFP-positive cells (green bars) and GFP-negative cells (gray bars; as an internal control). Data are mean±S.E.M. from four to six independent experiments

We found that the inducible Opa1_1–289_ protein was localized in the mitochondria, as shown both by confocal immunofluorescence microscopy (Figure 5C) and by subcellular fractionation (Figure 5B). Immunostaining with our Opa1 antibody recognizing both WT and the mutant Opa1 showed that the level of Opa1_1–289_ overexpression did not exceed twofold (*versus* endogenous normal Opa1) in most cells (Figure 6a). Despite the modest overexpression levels, Opa1_1–289_ -expressing cells apparently had a selective disadvantage, as we observed these cells being rapidly outgrown by a sub-population of non-expressing cells (Figure 6a).

To localize the Opa1 mutant, we used immunofluorescence microscopy. Surprisingly, the mutant Opa1 was distributed in small clusters associated with mitochondria, whereas endogenous Opa1 was distributed more uniformly throughout mitochondria (Figure 5C and Supplementary Figure S2). Figures 5C and D shows that Opa1_1–289_ expression did not affect mitochondrial elongation/fragmentation, as we observed that this protein was present in fused (tubular or elongated) mitochondria of normal lengths (typical for HeLa cells). Similarly, expression of the shorter (1–194) and longer (1–469) N-terminal-truncated mutants did not alter mitochondrial morphology. In contrast, overexpression of WT Opa1 resulted in mitochondrial fragmentation (Figures 5C and D), as has been observed in other studies.^6, 48^

To analyze the submitochondrial localization of the truncated Opa1 mutant protein, we used structural illumination microscopy (SIM). Image analysis (Supplementary Figure S2) displays the three-dimensional distance from fluorescent Opa1 speckles (either the mutant or WT) to the center of neighboring fluorescent speckles in Tim23-labeled mitochondria. The distance between the inner membrane marker Tim23 and Opa1_1–289_ was similar to that determined for Tim23 *versus* endogenous WT Opa1, which is known to be localized in the inner membrane.^48^ Also, Opa1_1–289_ was more proximal to Tim23 than to the outer membrane marker Tom20 as determined by comparison with Tom20-labeled mitochondria (Supplementary Figure S2). Additionally, the relative distance between Tom20 and Opa1_1–289_ was very similar to that determined for Tom20 *versus* Tim23 (Supplementary Figure S2). Taken together, the data indicate that Opa1_1–289_ is localized in the MIM, as expected.

Next, we tested whether Opa1_1–289_ had an effect on mitochondrial respiration in the Dox-induced cells. Although Opa1_1–289_ expression was typically unstable even at low expression levels, we were able to isolate clones maintaining Opa1_1–289_ expression in ~70–80% cells (as determined by FLAG immunostaining) for up to 2 days after Dox addition. We used these cells (derived from four individual clones) for bulk measurements of oxygen consumption in intact cells. Unlike the Opa1-haploinsufficient MEFs, Opa1_1–289_-expressing HeLa cells (which contain normal levels of endogenous WT Opa1) showed no decrease in mitochondrial respiration (Figure 5E). Furthermore, mitochondria containing the Opa1 mutants exhibited unaltered staining by the membrane potential-sensitive dye TMRE (not shown). Thus, we found no obvious effects of the Opa1 truncation mutants on mitochondrial morphology and function.

Finally, we asked whether Opa1_1–289_ expression altered the susceptibility of cells to apoptosis. Western blot analysis of whole-cell lysates showed that induction of apoptosis by certain agents caused a degradation of both mutant and normal Opa1 (Supplementary Figure S3). Because apoptotic cells lose at least some of their Opa1 content, we could not determine by simple immunostaining whether apoptotic cells had expressed FLAG-tagged Opa1 before mitochondrial permeabilization. Therefore, to quantify the effects of mutant Opa1 expression on apoptosis in heterogeneous cell populations containing both expressing and non-expressing cells, we used an IRES-containing bicistronic vector to express both the FLAG-tagged Opa1 mutant and green fluorescent protein (GFP), as a marker. Figure 6a confirms that Opa1_1–289_-expressing cells were both GFP- and FLAG-positive, whereas cells lacking the mutant were largely GFP-negative. Also, as expected, mitochondria in cells with higher GFP expression displayed a higher signal from immunostaining with the N-terminal Opa1 antibody (Figure 6a, bottom panels). After treatment of the cells with etoposide, we determined the percentage of apoptosis among GFP-positive and -negative cells by Bax immunostaining and confocal microscopy. Quite unexpectedly, we found that Opa1_1–289_-expressing cells showed less Bax activation than non-expressing (GFP-negative) or vector control cells (Figures 6b and c). We also observed increased resistance to etoposide-induced apoptosis in cells expressing the longer mutant Opa1_1–469_ (not shown), but not the shorter polypeptide Opa1_1–195_ lacking the coiled-coil domain (Figures 6b and c).

Previous reports have demonstrated that Opa1 has a role in apoptosis by controlling a mitochondrial inner membrane-remodeling step (crista junction opening) necessary for the release of mitochondrial proteins from cristae.^8, 9^ We showed that this Opa1-dependent crista junction remodeling is Bax/Bak-dependent, downstream of Bax activation, and independent of MOMP.^8^ In contrast to that event, in which Bax and Bak activation signals inward to Opa1, here we describe a different situation in which an N-terminal fragment of Opa1 can signal outward to inhibit MOM permeabilization, possibly by interacting with another mitochondrial protein. As the N-terminal membrane-integration domain is required for this effect, we considered Higd1a, an inner membrane protein interacting with the N-terminal portion of Opa1, as a candidate.^49^ However, we found that siRNA silencing of that protein had no effect on etoposide-induced apoptosis (not shown). It remains to be investigated how the signal from native Opa1 (or its mutant forms) in the inner membrane is conveyed to the MOM or upstream of MOMP.

Thus, the overexpression of mitochondria-targeted Opa1 truncation mutants causes no obvious deleterious effects such as mitochondrial dysfunction or increased cell death. In any case, our data rule out the expression of a truncated Opa1 mutant in the Opa1^Q285STOP^ mouse ADOA model, allowing us to conclude that the ADOA-like phenotype results entirely from haploinsufficiency. This suggests that the effects of similar Opa1-truncating mutations in humans may also arise from Opa1 haploinsufficiency, rather than a dominant effect of mutant Opa1 protein. As we showed, haploinsufficiency in the mice results in defects in mitochondrial respiration and COX expression. We propose that these defects could lead to metabolic stress in certain vulnerable cell types such as RGCs or cardiac muscle.

## Materials and Methods

### Animals and MEF preparation

Heterozygous Opa1^Q285STOP^ mice (on the C57Bl/6 background) were generated previously.^20^ Mouse colony maintenance and all animal work were approved by the Institute's Animal Care and Use Committee. Genotyping was performed at Transnetyx (Cordova, TN, USA). Primary cultures of MEFs were generated as described.^33^ Briefly, embryos were collected in individual dishes from E13.5–E15.5 Opa1^Q285STOP^ mice, dissected, and incubated in 0.25% trypsin-EDTA solution at 4 °C overnight. The next day, the tissue was dissociated and cell suspension obtained from each embryo was plated in five to six 10-cm dishes with DMEM (Life Technologies, Carlsbad, CA, USA) containing penicillin and streptomycin (100 U/ml and 1000 *μ*g/ml, respectively) and 10% fetal bovine serum (FBS; Gemini Bio Products, West Sacramento, CA, USA). Opa1 mutant and the littermate control (WT) MEFs were expanded during the rapid proliferation at early passages and maintained in the culture up to 10–15 passages. Immortalized *opa1*-null and control MEFs were obtained from ATCC (Manassas, VA, USA).

### Production of antibody against N-terminal portion of Opa1 (114–289)

Truncated Opa1 (114–289) in pET28 (no FLAG-tag) was expressed in BL21(DE3) cells. The protein was solubilized from the inclusion bodies in 8 M urea, purified with Ni^2+^-NTA agarose (Qiagen, Hilden, Germany) and used to immunize rabbits. Opa1 antiserum was produced using a 13-week antibody production protocol at Pacific Immunology (Ramona, CA, USA). Specific immunoglobulin for Opa1(114–289) was affinity-purified using recombinant Opa1(114–289) protein coupled to Sepharose as follows. Bacterially expressed Opa1 (114–289) was solubilized in phosphate-buffered saline (PBS) containing 1% Triton X-100 and purified on Ni^2+^-NTA agarose. The protein (2.7 mg) was coupled to 1.35 ml CNBr-Sepharose (GE Healthcare Bio-Science, Piscataway, NJ, USA) according to the manufacturer's instructions. Ten milliliters of the antisera from later bleeds were incubated with the truncated Opa1-coupled Sepharose overnight at 4 °C. The beads were washed and eluted with 0.1 M glycine-HCl, pH 3.0, 0.5 M NaCl, and the eluted fractions were immediately neutralized with 1 M Tris, pH 8. The peak immunoglobulin fractions were collected, dialyzed in PBS, and concentrated to 0.85 mg/ml.

### Western blot analysis

For analysis of Opa1 protein in different tissues, samples of retina, brain, spleen, and liver were collected from aged (>12- month-old) Opa1 mutant and littermate WT animals. Livers were used for isolation of mitochondria by differential centrifugation.^50^ In some experiments, tissue samples and liver mitochondria were isolated from younger (3-month-old) mice. For immunoblotting, tissue lysates or isolated mitochondria were loaded on NuPage 4–12% Bis-Tris gels (Life Technologies) at 30–50 *μ*g per lane. For western blot analysis of Opa1 and other proteins in cultured cells, cell aliquots (~0.5–1 million) were centrifuged at a low speed and the pellets were resuspended in 50–100 *μ*l ice-cold lysis buffer containing 0.5% Nonidet P-40, 50 mM Tris-HCl (pH 8.0), 150 mM NaCl and 1 × Complete protease inhibitor mixture (Roche Biochemicals, Indianapolis, IN, USA). After 20 min incubation on ice, whole-cell lysates were centrifuged at 15 000x*g* for 15 min. Supernatants were used for the analysis. For subcellular fractionation, cells inducibly expressing Opa1 mutant or vector control were permeabilized with digitonin (0.008% per 3 millions cells) in 0.5 ml buffer containing 125 mM KCl, 2 mM KH_2_PO_4_, and 20 mM HEPES-KOH (pH 7.4). Cytosolic and membrane (mitochondria-enriched) fractions were separated by centrifugation at 15 000x*g* for 3 min at 4 °C. Whole-cell extracts or subcellular fractions were loaded on the gels at 30–40 *μ*g per lane. Protein concentrations were determined by Pierce BCA protein assay (Thermo Scientific, Waltham, MA, USA). Gels were run at 200 V for 45 min. Proteins were electrotransferred to nitrocellulose membrane (Bio-Rad, Hercules, CA, USA) at 30 V for 75 min. The membranes were blocked in 2.5% milk overnight at 4 °C and probed with the custom-made rabbit Opa1 antisera. Other antibodies used were: VDAC (porin) (anti-mouse from Calbiochem, Billerica, MA, USA; anti-rabbit from Abcam), cytochrome *c* (BD Pharmingen; BD Biosciences, San Jose, CA, USA), COX subunits I and II (Molecular Probes, Eugene, OR, USA) and COX Va (Abcam), Hsp60 (Santa Cruz Biotechnology, Dallas, TX, USA), NDUFB8 (Novex, Life Technologies), NDUFS7 (20 kDa subunit) (Molecular Probes), Drp1(Dnm1) (BD Transduction Laboratories, Eugene, OR, USA), Tom20 (Santa Cruz Biotechnology), and Bax(N20) (Santa Cruz Biotechnology). A mouse Opa1 antibody (BD Transduction Laboratories) was used for the immunoblot shown in Figure 1a. Anti-FLAG(M2) antibody was from Sigma (St. Louis, MO, USA). Secondary antibodies used were horseradish peroxidase-conjugated anti-mouse and anti-rabbit antibodies (Amersham, Amersham, UK), or an anti-goat antibody (Santa Cruz Biotechnology). Protein bands were detected using either SuperSignal WestPico or SuperSignal West Femto reagent (Thermo Scientific) and X-ray film.

### Generation of Tet-on-inducible HeLa cells

We used a Tet-on gene expression system to induce expression of indicated Opa1 mutant proteins (Figure 5A). Each mutant or WT Opa1 was PCR-amplified and inserted at the multiple cloning site of pTRE2hyg (Clontech, Mountain View, CA, USA) that contained a tetracycline-responsive promoter. We also constructed pTRE2hyg-OpaI with IRES-GFP, to monitor the induction of the protein. PCR-amplified IRES-GFP was inserted downstream of Opa1. All the constructs were verified by sequencing. The cells were transfected with Lipofectamine 2000 (Life Technologies) and stably expressing cells were selected in the presence of G418 (200–400 *μ*g/ml) and hygromycin B (200–400 *μ*g/ml) (Gemini Bio Products). Opa1_1–289_-IRES-GFP cells were sorted to enrich the population with the Opa1_1–289_-expressing (GFP-positive) cells. Cells were maintained in DMEM containing 10% Tet system-approved FBS (Clontech), 100 *μ*g/ml G418, and hygromycin B. The medium was changed every 3–4 days. Experiments were performed 1–2 days after induction with Dox (1 *μ*g/ml).

### Immunostaining, confocal and super-resolution microscopy

For fluorescent microscopy, cells were grown on 35 mm glass bottom MatTek dishes (MatTek Corporation, Ashland, MA, USA) or glass coverslips no. 1.5 (Corning, Corning, NY, USA) placed in 6-well plates. Cells were fixed with 0.5% glutaraldehyde diluted in PBS from an 8% stock solution (Sigma) for 40 min at 4 °C. Autofluorescence was quenched by 0.5% freshly prepared sodium borohydride at room temperature for 30 min. Cells were permeabilized with 0.5% Triton X-100 in PBS (15 min), followed by 1 h incubation in blocking buffer containing 2% bovine serum albumin, 0.05% Tween-20, and 0.1% NaN_3_ in PBS before the addition of primary antibodies. The antibodies used were: Bax(N20) and Tom20 (FL-145) from Santa Cruz Biotechnology Inc.; mouse cytochrome *c* (BD Pharmingen), Tim23 (BD Biosciences), Bax (6A7) (TACS), FLAG(M2) from Sigma or Alexa-647-conjugated FLAG (Cell Signaling, Danvers, MA, USA). The antibodies were diluted at 1 : 500 in the blocking buffer; Tom20 antibody dilution was 1 : 1000. Samples were incubated with primary antibodies for 1–2 h at RT, washed several times with PBS, and incubated in the blocking buffer for 30 min before the addition of secondary fluorophore-conjugated antibodies (usually, diluted 1 : 500 in the blocking buffer) for 1.5–2 h at RT. The secondary antibodies used were: rabbit or mouse Alexa-488, Alexa-568, and Alexa-647 (Life Technologies). Nuclei were stained with Hoechst 33342 (1 : 10 000 dilution) and washed in PBS. Samples prepared for super-resolution microscopy (below) were additionally postfixed in 4% paraformaldehyde in PBS. Stacks of confocal images were acquired with a x60 (1.3 na) oil immersion objective on an Olympus FluoView FV10i automated confocal laser scanning microscope (Olympus Scientific Solutions America Corp, Waltham, MA, USA). Images reconstructed in 3D (Figure 5C, panels a–d) were generated on a Nikon A1R confocal super-resolution system (Mellville, NY, USA) using a x100 (1.46 na) oil immersion objective. Stacks of z-series images were collected using a 0.1 *μ*m z step size. Images were then further rendered and processed in the IMARIS 3D modeling software (Bitplane-Andor Inc., an Oxford Instruments Co, Concord, MA, USA).

In imaging experiments involving quantification of cells with different phenotypes, 100–300 cells in each experiment (*n*≥3) were blindly counted for each sample. The percentage of cells with activated Bax was determined based on Bax(N20) antibody staining. In some experiments, cells were costained with cytochrome *c* antibody to confirm that cytochrome *c* was released from the mitochondria in apoptotic cells (i.e., cells with activated Bax). The concentrations of apoptogens are indicated in figure legends. All apoptogens were added in the presence of caspase inhibitor Q-VD (Q-VD-OPH, SM Biochemicals LLC, Anaheim, CA, USA). In the absence of added apoptogens, the number of cells showing Bax(N20) staining was negligibly small. Quantification of the cells with different mitochondrial morphologies (e.g., tubular or fragmented) was based on immunostaining with mitochondrial markers (cytochrome *c*, Tom20, or Tim23).

#### Super-resolution microscopy

Images were also acquired using either a Zeiss SIM ELYRA S13D Super-resolution Microscope or a Zeiss 880 laser scanning confocal microscope with Airy Scan super-resolution. Z-series image stacks of multilabeled samples immunostained for Tom20, Tim23, and Opa1 were optically acquired with a x63 (1.4 na) objective using a 0.10 *μ*m step size and then processed using ZEN software (Zeiss Inc., Thornwood, NY, USA). Further processing and rendering of images was completed using the IMARIS software (Bitplane-Andor Inc.). In IMARIS, fluorescent signals were iso-surfaced into 3D objects to create distance transform maps (MATLAB macro) of the objects defining their spatial relationship to each other. Once the objects were defined, Opa1-positive 3D signal objects were compared with either Tom20- or Tim23-labeled 3D objects, and the final distance transform image displayed is shown as a color-coded distance map: green-red – <140 nm; purple blue – >140 nm (see Supplementary Figure S2). Additionally, the distance map was generated for Tim23 *versus* Tom20 for comparison (Supplementary Figure S2, bottom panels). Numbers shown in the figures represent an averaged percentage±S.E.M. of the total number of objects per cell within the shortest distance between objects. An average of three cells per group was analyzed.

### Respiration (oxygen consumption) measurements and flow cytometry

Respiration of intact MEFs was measured with Seahorse Bioscience Inc. (Billerica, MA, USA) XF24, XF96, or XFe96 Flux Analyzers. Cells were plated on the Seahorse cell culture plates in their growth medium at a density of 2 × 10^4^ or 10^4^ cells per well for 24- and 96-well plates, respectively, 24 h before the measurement. For apoptogen testing, cells were plated 48 h before the experiment at a density of 10^4^ or 5 × 10^3^ cells per well for 24- and 96-well plates, respectively. At times indicated in the Figure legends, the medium was supplemented with an equal volume of the same medium containing 2 × concentrations of various apoptogens. Immediately before measurement the medium was removed, and the cells were gently washed with assay buffer (unbuffered DMEM prepared according to Seahorse protocols and supplemented with 10 mM glucose, 10 mM sodium pyruvate and 1 × GlutaMax, pH 7.4). Wells were filled with 450 or 100 *μ*l of assay buffer for 24- and 96-well plates, respectively, and the measurements were performed in the Seahorse apparatus according to the manufacturer's recommendations. Enzymatic activity of mitochondrial Complex IV was measured in cells permeabilized with 3 nM perfringolysin O (PMP; Seahorse Biosciences) in MAS-1 medium (Seahorse Biosciences) supplemented with 0.2% BSA (bovine serum albumin). Complex IV activity was assayed as oxidation of its artificial electron donor TMPD, as measured by the OCR. For these experiments, 10^5^ MEFs per well (96-well plate) were seeded 24 h before the assay. ADP (4 mM) and succinate (10 mM) with rotenone (2 *μ*M) were added to the medium to relieve respiratory control and place mitochondria in the metabolic state 3. Maximal activity of Complex IV was measured after inhibiting the upstream portion of the respiratory chain with Complex III inhibitor antimycin A, followed by addition of TMPD (in the presence of ascorbic acid to keep it re-reduced). The specificity of the reaction was then verified using Complex IV inhibitor azide (20 mM). The mitochondrial membrane potential in MEFs was evaluated by TMRE staining and flow cytometry as described.^42^ Respiration of intact Dox-induced cells in suspension was measured using a Clark-type electrode (Hansatech, King's Lynn, UK) as described.^42^ Briefly, vector control or Opa1_1–289_-expressing cells were resuspended in DMEM (10 million cells per ml) and basal (no additions), state 4 (oligomycin-induced) and state 3u (FCCP-induced) respiration rates were measured in a ~20 min run. FCCP and oligomycin concentrations were 300 nM and 2 *μ*g/ml, respectively.

### Statistical analyses

Unless noted otherwise, two-way analysis of variance (ANOVA) with *post hoc* Bonferroni tests was performed using GraphPad Prism software (La Jolla, CA, USA). Symbols: ***P<0.001; **P<0.01; *P<0.05.

## Abbreviations

ADOA: autosomal dominant optic atrophy
Opa1: optic atrophy 1
RGC: retinal ganglion cell
MOM: mitochondrial outer membrane
MOMP: mitochondrial outer membrane permeabilization
WT: wild type
MEF: mouse embryonic fibroblast
COX: cytochrome *c* oxidase
ER: endoplasmic reticulum
OCR: oxygen consumption rate
Mfn1: Mitofusin 1
Mfn2: Mitofusin 2
ETC: electron transport chain
FCCP: 4-(trifluoromethoxy) phenylhydrazone carbonyl cyanide
STS: staurosporine
mtDNA: mitochondrial DNA
GFP: green fluorescent protein
Dox: doxycycline
DMEM: Dulbecco's modified Eagle's medium
PBS: phosphate-buffered saline
FBS: fetal bovine serum
SIM: structural illumination microscopy
