# Supplementary material for: Mitochondrial dysfunction in an Opa1Q285STOP mouse model of dominant optic atrophy results from Opa1 haploinsufficiency
Source: Cell Death Dis. 2016 Jul 28;7(7):e2309–. doi: 10.1038/cddis.2016.160 (PMC4973340; doi:10.1038/cddis.2016.160)
Supplement: Supplementary Figure Legends [file cddis2016160x4.doc]

# Figure Legends

**Supplementary Figure 1. A.** Mitochondrial respiration (OCR) measured in WT and Opa1^Q285STOP^ MEFs permeabilized with perfringolysin O (PFO). State 3 respiration was achieved with 4 mM ADP. State 4 (oligomycin-inhibited) and State 3u (FCCP-induced) OCR were measured as described in Fig. 2C for intact cells. Data shown are mean ± SE from 3 experiments. **B.** Complex IV level was reduced in Opa1^Q285STOP^ MEFs. Western Blot analysis of Complex IV subunits (Cox I and COX II) in whole cell lysates prepared from independent cultures of WT and Opa1 mutant MEFs. Hsp60 is shown as a loading control. The blot on the right (displayed at two exposure times) also includes a sample from Opa1 null MEFs for comparison.

**Supplementary Figure 2**. Analysis of mitochondrial localization of Opa1_1-289_ mutant (Opa1-FLAG) by superresolution microscopy. Doxycycline-induced cells expressing Opa1_1-289_ were immunostained with FLAG and either Tim23 or Tom20 antibodies as indicated. Uninduced (non-expressing) cells were stained with Opa1 and Tim23 antibodies (upper panels) for comparative analysis of endogenous WT Opa1. Bottom panels also show Tim23 vs. Tom 20 analysis. Opa1 localization relative to the inner membrane (Tim23) or the outer membrane (Tom20) markers is shown as a color-based distance map (green-red – less than 140nm; blue-purple - greater than 140nm) depicted in each panel. Numbers indicate the percentage +/-SEM of the total number of objects per cell within the shortest distance between objects (color coded green-red - less than 140nm). An average of 3 cells per group was analyzed.

**Supplementary Figure 3**. Effects of apoptogens on the level of WT and mutant Opa1. Western blot analysis (shown in duplicate) of whole cell lysates prepared from Dox-induced cells expressing either FLAG-tagged Opa1_1-289_ or FLAG-tagged Opa1_1-469_ and treated with 300 μM etoposide or 10 μM actinomycin D (Act D) in the presence of 20 μM Q-VD for indicated time (h). Both apoptogens induce variable degrees of degradation of endogenous Opa1 and Opa1 mutants. The levels of Opa1 mutants are also shown at a higher exposure time (bottom panel). Opa1 mutants were detected with FLAG antibody. Hsp60 is shown as a loading control.
